# The effects of psychological distress and its interaction with socioeconomic position on risk of developing four chronic diseases

**DOI:** 10.1016/j.jpsychores.2018.04.004

**Published:** 2018-06

**Authors:** Kyle J.J. McLachlan, Catharine R. Gale

**Affiliations:** a Edinburgh Medical School, College of Medicine and Veterinary Medicine, University of Edinburgh, Edinburgh, UK; b Department of Psychology, University of Edinburgh, Edinburgh, UK; c Centre for Cognitive Ageing and Cognitive Epidemiology, Department of Psychology, University of Edinburgh, Edinburgh, UK; d MRC Lifecourse Epidemiology Unit, University of Southampton, Southampton, UK

**Keywords:** Psychological distress, Depression, Arthritis, Cardiovascular disease, Chronic obstructive pulmonary disease, Diabetes, BMI, Body Mass Index, COPD, Chronic Obstructive Pulmonary Disease, CVD, Cardiovascular disease, GHQ, General Health Questionnaire, OR, Odds Ratio, SEP, Socioeconomic Position

## Abstract

**Objective:**

To examine the relationship between psychological distress and risk of developing arthritis, cardiovascular disease, chronic obstructive pulmonary disease and diabetes across the range of distress severity, investigate the mediating roles of health behaviours and explore whether the associations vary with socioeconomic position.

**Methods:**

Participants were 16,485 adults from the UK Household Longitudinal Study We examined prospective relationships between psychological distress at baseline (measured using the 12-item General Health Questionnaire) and incidence of arthritis, cardiovascular disease, chronic obstructive pulmonary disease and diabetes (measured using self-report) over 3 years using logistic regression. We then examined the mediating effects of health behaviours and investigated whether the associations varied with socioeconomic position.

**Results:**

Distress significantly increased risk of incident arthritis, cardiovascular disease and chronic obstructive pulmonary disease in a dose-response pattern after controlling for age, sex, socioeconomic position, neighbourhood cohesion, marital status, BMI and baseline disease. High levels of distress (GHQ ≥ 7) increased risk of arthritis (OR 2.22; 1.58–2.13), cardiovascular disease (OR 3.06; 1.89–4.98) and chronic obstructive pulmonary disease (OR 3.25; 1.47–7.18). These associations were partially mediated by smoking status but remained significant after controlling for smoking status, diet and exercise. Distress significantly predicted incident diabetes in manual socioeconomic groups only. Effect sizes did not vary with socioeconomic position for arthritis, cardiovascular disease and chronic obstructive pulmonary disease.

**Conclusion:**

Psychological distress increases risk of incident arthritis, cardiovascular disease and chronic obstructive pulmonary disease in a dose-response pattern, even at low and moderate distress levels. Future research should investigate the mediating role of inflammatory biomarkers.

## Highlights

• Increasing levels of psychological distress raises risk of future chronic disease.
• Risk of arthritis, lung disease and cardiovascular disease rose as distress increased.
• Smoking habits explained a small part of these associations.

## 1 Introduction

Clinical depression and anxiety have been linked with the development of a variety of chronic diseases. There is evidence from several longitudinal studies that depression and anxiety increase risk of incident arthritis [1,2], cardiovascular disease (CVD) [[3], [4], [5], [6], [7], [8]], chronic obstructive pulmonary disease (COPD) [1,9] and diabetes mellitus [[10], [11], [12]]. However, the health effects of less severe symptoms of depression and anxiety are poorly understood.

The symptoms of depression and anxiety are collectively termed psychological distress. Psychological distress encompasses a much wider range of experiences than mental illness, ranging from mild symptoms to severe psychiatric disease [13]. The 12-item General Health Questionnaire (GHQ-12) [14,15] is commonly used to measure psychological distress in population studies [16]. Clinically significant levels of distress (i.e. a GHQ-12 score of four or greater [14,17]) have been found to increase risk of incident CVD [18], COPD [19] and diabetes [20]. To our knowledge, no study to date has used GHQ-12 scores to examine the relationships between psychological distress and incidence of chronic diseases across the whole range of distress severity (i.e. comparing the effects of subclinical, moderate and high distress).

Recent studies have found a dose-response relationship between psychological distress and risk of mortality from all causes [21,22], colorectal and prostate cancers [23] and CVD [24,25] across the whole range of distress severity. Chronic diseases, and particularly cardiovascular diseases, are undoubtedly the most common causes of all-cause mortality in these studies [26]. It is therefore plausible that the risk of developing chronic disease will increase with increasing levels of distress severity in a similar fashion.

Lazzarino et al. [22,25] found that the effects of distress on mortality were stronger in people with lower socioeconomic position (SEP). It is unclear why psychological distress has greater consequences for health in people with low SEP. Investigating whether distress and SEP also interact to increase risk of incident chronic diseases and examining potential mediators of any associations will improve understanding of the relationship between psychological distress, SEP and health.

In this study, we will focus on arthritis, COPD, CVD and diabetes because although the relationship between significant distress and onset of these conditions is well-established [1,4,7,9,10], the impact of subclinical levels of distress on disease incidence remains a significant gap in knowledge. The evidence is less conclusive for other common conditions such as cancer [21,23,27] and liver disease [28] and further research into the effects of significant psychological distress on incidence of and mortality from these diseases is required before investigating the effects of lower levels of distress.

We used data from the UK Household Longitudinal Study (UKHLS) to investigate the prospective relationships between psychological distress and incidence of four chronic diseases (arthritis, CVD, COPD and diabetes mellitus) in participants aged 18 and over. We then examined whether the strengths of these associations varied with SEP category. In both analyses, we controlled for age, sex, SEP, marital status, neighbourhood cohesion, body mass index and chronic disease at baseline and then examined the potential mediating roles of unhealthy behaviours (smoking, poor diet and physical inactivity).

## 2 Methods

The UK Household Longitudinal Study (UKHLS) is a stratified clustered random sample of households representative of the United Kingdom population. It began in 2009 and gathers data annually from a population sample selected from 39,802 UK households [29]. All members of selected households over the age of ten are included in the study, amounting to 101,086 participants from diverse socioeconomic and ethnic backgrounds [29]. Data on health, psychological, social and economic variables is gathered once a year from adults using interviews and written questionnaires. In the current study we used data from the General Population Sample from wave 1 (2009–2010), wave 2 (2010−2011) and wave 3 (2011−2012) [30]. Ethical approval was granted by the University of Essex Ethics Committee. Participants gave written informed consent.

## 3 Measures

### 3.1 Incident disease

Participants were interviewed to find out whether they had been diagnosed with chronic disease at baseline using the question: “Has a doctor or other health professional ever told you that you have any of the conditions listed on this card?” At waves 2 and 3, participants were asked to report any newly diagnosed conditions from the same list. For the purpose of our analyses, we grouped the diagnoses of coronary heart disease, myocardial infarction, angina and stroke under the category of cardiovascular disease (CVD) and bronchitis and emphysema under the category of COPD. Incident disease variables were created for arthritis, COPD, CVD and diabetes by coding participants who reported a new diagnosis of the condition at wave 2 or wave 3 as “1” and participants who did not report a new diagnosis of the specific condition as “0”. These four incident disease variables were used as the main outcomes in our analyses.

### 3.2 Predictors

Psychological distress at baseline was measured using the 12-item version of the General Health Questionnaire (GHQ) [14]. The GHQ is used frequently in population studies to measure the extent to which individuals experience symptoms of depression, anxiety and other negative mental health states [31]. Participants are asked to indicate how frequently they experience 12 common symptoms (e.g. loss of sleep, loss of confidence). We recoded the response scores for each item using the bimodal method used by other researchers [21,22] as follows: 0 = ‘*not at all’*, 0 = ‘*no more than usual*’, 1 = ‘*more than usual’*, 1 = ‘*much more than usual*’. Using this method of interpretation, participants with a total GHQ-12 score of four or greater are considered to be a case of psychological distress. This threshold has been validated against standard psychiatric interviews and was found to correspond to clinical depression [17]. In order to investigate the effects of distress across the whole range of symptom severity, we divided total GHQ scores into four distinct groups: “*asymptomatic*” (0), “*low distress*” [[1], [2], [3]], “*moderate distress*” [[4], [5], [6]] and “*high distress*” [[7], [8], [9], [10], [11], [12]]. Russ et al. [21] categorised GHQ scores using the same four groups.

Socioeconomic position (SEP) was measured at baseline using occupation. Each participant's current job, or most recent job for unemployed participants, was categorised according to Registrar General's Social Class. There were six categories of occupational social class: *“professional occupation”* [1], *‘managerial and technical occupation’* [2], *“skilled non-manual”* [3], *“skilled manual”* [4], *“semi-skilled occupation”* [5] and “*unskilled occupation”* [6]. For the purposes of analysing the interaction between distress and SEP, we recoded these occupational classes into two broad categories: 1 = “*non-manual*” [[1], [2], [3]] and 2 = “*manual*” [[4], [5], [6]].

### 3.3 Potential mediators

The potential mediators included in analyses were smoking status, diet and exercise, all measured at wave 2. Smoking status was measured by asking participants “Have you ever smoked a cigarette, a cigar or a pipe?” (ever smoked) and “Do you smoke cigarettes at all nowadays?” (current smoker). The responses to these two questions were recoded to create a smoking status variable, where 0 = “*never smoked*”, 1 = “*ex-smoker”* and 2 = “*current smoker*”. Level of exercise was measured by asking participants “On how many days in the last four weeks did you spend 30 minutes or more walking?” Quality of diet was measured by asking participants “On a day when you eat fruit or vegetables, how many portions of fruit and vegetables in total do you usually eat?”

### 3.4 Other covariates

Age, sex, body mass index (BMI), chronic disease at baseline, SEP, and social support, as indicated by marital status and neighbourhood cohesion at wave 1, were included as potential confounding variables in analyses. Neighbourhood cohesion was measured at baseline using eight items form the ‘Neighbourhood Cohesion Scale’ [32] which assesses the availability and quality of local social support [33]. Participants were asked to indicate the extent to which they agree with statements (e.g. “I talk regularly to my neighbours”) on a 5-point Likert scale ranging from 1 = “*strongly disagree*” to 5 = “*strongly agree*”. This eight-item questionnaire has been found to be unidimensional and have high levels of internal consistency (α = 0.87) [34]. Item scores were added to give a total score for neighbourhood cohesion.

## 4 Statistical methods

Our analyses were conducted using 16,485 participants aged 18 or over who had complete data on all variables. This sample amounts to 41.7% of the 39,573 people aged 18 and older who participated in the study between waves 1 and 3.

We used binary logistic regression to investigate the relationships between psychological distress at baseline and incidence of arthritis, CVD, COPD and diabetes. Participants diagnosed as having the disease of interest at Wave 1 were excluded from the analysis. The disease risks associated with low, moderate and high levels of distress were examined using asymptomatic participants as a reference group. Conducting analyses with men and women separately led to a very low number of disease events in some groups so we analysed men and women together, adjusting for age and sex (Model 1), further adjusting for SEP, neighbourhood cohesion and marital status (Model 2), and then adding BMI and other chronic diseases at baseline to the model (Model 3). We further adjusted for diet, exercise and smoking status (Model 4) and used Sobel-Goodman and boot-strapping tests to examine whether or not these health behaviours were significant mediators. 25% of the participants who had complete data on incident disease were excluded from our analyses because of missing data on one or more of the covariates. In order to investigate whether excluding these cases biased the results of this study [35], we carried out multiple imputation for missing covariate data on cases with compete incident disease data and repeated analyses with the 18 imputed data sets that we generated. The proportion of imputed values ranged from 1.78% for smoking status to 17.35% for exercise. Finally, we investigated whether the effect of psychological distress on incidence of arthritis, CVD, COPD and diabetes varied with SEP after controlling for age, sex, BMI and chronic disease at baseline. Statistical analyses were carried out using SPSS (Statistical Package for the Social Sciences) for windows (v. 22.0.0.1) and STATA (v. 14).

## 5 Results

Table 1 shows the characteristics of the sample according to psychological distress. Higher levels of psychological distress were significantly associated with higher BMI, poorer health behaviour (in terms of diet, exercise and smoking), lower SEP and lower neighbourhood cohesion. People with high levels of psychological distress were also significantly more likely to be younger, female and married or living with a partner and have arthritis and COPD at baseline. During the 3-year follow-up period, 410 (2.9%) participants developed arthritis, 173 (1.1%) developed cardiovascular disease, 55 (0.3%) developed COPD and 141 (0.9%) developed diabetes.

**Table 1:** Characteristics of the sample according to level of psychological distress (n = 16,485).

Characteristics	Level of psychological distress				p value
	Asymptomatic (n = 9482)	Low (n = 4362)	Moderate (n = 1346)	High (n = 1295)	
Age (years), mean (SD)	50.42 (15.75)	46.94 (15.99)	45.77 (15.57)	45.61 (13.39)	<0.001
BMI (kg/m^2^), mean (SD)	26.20 (4.58)	26.50 (4.99)	26.23 (5.11)	26.82 (5.86)	<0.001
Diet^a^, mean (SD)	3.53 (1.56)	3.40 (1.52)	3.34 (1.49)	3.25 (1.55)	<0.001
Exercise^b^, mean (SD)	11.01 (9.21)	10.28 (9.97)	9.86 (9.87)	9.80 (10.16)	<0.001
Neighbourhood cohesion, mean (SD)	29.49 (5.85)	28.69 (6.19)	27.96 (6.55)	27.78 (6.91)	<0.001
Female, %	54.00	56.95	63.60	63.63	<0.001
SEP^c^, %					<0.001
Unskilled	4.32	4.08	4.98	4.94	–
Semi-skilled	13.32	14.44	16.05	17.06	–
Skilled manual	17.74	17.54	16.72	18.38	–
Skilled non-manual	21.69	22.24	23.85	26.02	–
Managerial/technical	36.54	35.30	33.88	29.11	–
Professional	6.38	6.40	4.52	3.78	–
Baseline disease, %					
Arthritis	12.19	15.45	16.49	17.92	<0.001
COPD^d^	1.69	2.66	2.45	4.17	<0.001
Diabetes	4.69	4.61	4.75	5.56	0.544
CVD^e^	4.90	5.55	5.87	6.33	0.068
Smoking status, %					<0.001
Never smoked	41.36	40.97	36.70	33.36	–
Ex-smoker	41.47	38.70	37.89	35.14	–
Current smoker	17.17	20.33	25.41	31.51	–
Married or living with a partner, %	24.85	28.66	33.36	39.54	<0.001

Note: p value indicates significance of test for linear trend in continuous variables and significance of chi-square test for categorical variables.

a Number of portions of fruit and vegetable eaten in a day.

b Number of days in the last four weeks on which participants spent ≥30 min walking.

c Socioeconomic position as indexed by occupation.

d Chronic obstructive pulmonary disease.

e Cardiovascular disease.

**Fig. 1:**
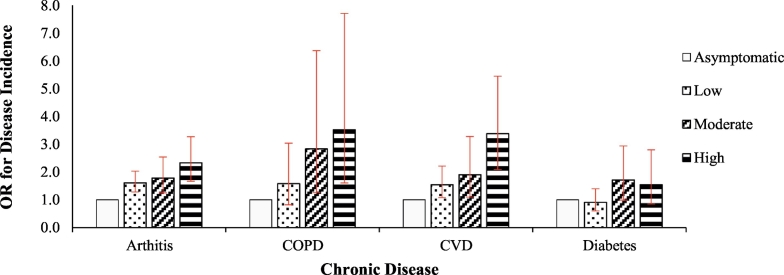
Age- and sex- adjusted odds ratios (ORs) for disease incidence associated with different levels of psychological distress.

Table 2 shows the odds ratios for incident disease associated with different levels of psychological distress compared to asymptomatic participants. After adjusting for age and sex, there were significant associations between psychological distress and risk of arthritis and CVD across the full range of distress severity. Risk of developing COPD was also significantly associated with moderate and high levels of psychological distress but not low levels of distress. The linear trends between level of psychological distress and risk of arthritis, CVD and COPD were significant in all models (Table 2), indicating dose-response relationships between distress and risk of developing these conditions (Fig. 1). There were no significant associations between psychological distress and risk of diabetes.

**Table 2:** Odds ratios (95% CI) for incident chronic disease according to level of psychological distress (n = 16,485).

Chronic disease	Level of psychological distress				p-value for linear trend
	Asymptomatic n = 9482	Low n = 4362	Moderate n = 1346	High n = 1295	
Arthritis	n = 206	n = 120	n = 39	n = 45	
Model 1^a^	**1.00**	**1.61** (1.28–2.03)⁎⁎	**1.78** (1.25–2.54)⁎⁎	**2.33** (1.67–3.27)⁎⁎	<0.001
Model 2^b^	**1.00**	**1.62** (1.28–2.04)⁎⁎	**1.79** (1.25–2.55)⁎⁎	**2.30** (1.64–3.24)⁎⁎	<0.001
Model 3^c^	**1.00**	**1.58** (1.25–2.00)⁎⁎	**1.78** (1.25–2.54)⁎⁎	**2.22** (1.58–2.13)⁎⁎	<0.001
Model 4^d^	**1.00**	**1.57** (1.24–1.99)⁎⁎	**1.72** (1.20–2.46)⁎⁎	**2.11** (1.50–2.98)⁎⁎	<0.001
CVD	n = 86	n = 48	n = 16	n = 23	
Model 1^a^	**1.00**	**1.54** (1.08–2.21)⁎	**1.90** (1.10–3.28)⁎	**3.38** (2.09–5.45)⁎⁎	<0.001
Model 2^b^	**1.00**	**1.53** (1.07–2.19)⁎	**1.88** (1.09–3.25)⁎	**3.19** (1.97–5.17)⁎⁎	<0.001
Model 3^c^	**1.00**	**1.45** (1.01–2.09)⁎	**1.80** (1.04–3.13)⁎	**2.98** (1.83–4.85)⁎⁎	<0.001
Model 4^d^	**1.00**	**1.46** (1.02–2.10)⁎	**1.77** (1.02–3.08)⁎	**2.89** (1.77–4.74)⁎⁎	<0.001
COPD	n = 23	n = 15	n = 8	n = 9	
Model 1^a^	**1.00**	**1.58** (0.82–3.04)	**2.83** (1.26–6.37)⁎	**3.52** (1.61–7.71)⁎⁎	<0.001
Model 2^b^	**1.00**	**1.52** (0.79–2.92)	**2.54** (1.12–5.76)⁎	**2.99** (1.35–6.60)⁎⁎	0.002
Model 3^c^	**1.00**	**1.48** (0.77–2.87)	**2.50** (1.10–5.69)⁎	**2.91** (1.31–6.47)⁎⁎	0.003
Model 4^d^	**1.00**	**1.44** (0.74–2.78)	**2.25** (0.99–5.15)	**2.48** (1.11–5.56)⁎	0.011
Diabetes	n = 82	n = 30	n = 16	n = 13	
Model 1^a^	**1.00**	**0.91** (0.60–1.40)	**1.71** (0.99–2.94)	**1.54** (0.85–2.80)	0.07
Model 2^b^	**1.00**	**0.91** (0.60–1.39)	**1.68** (0.98–2.90)	**1.48** (0.81–2.70)	0.09
Model 3^c^	**1.00**	**0.83** (0.54–1.27)	**1.58** (0.91–2.75)	**1.24** (0.67–2.29)	0.30
Model 4^d^	**1.00**	**0.83** (0.54–1.27)	**1.52** (0.87–2.65)	**1.21** (0.65–2.24)	0.36

⁎ p < .05.

⁎⁎ p < .01.

a Adjusted for age and sex.

b Further adjusted for SEP (wave 1), neighbourhood cohesion (wave 1) and marital status (wave 1).

c Further adjusted for BMI (wave 1) and other chronic diseases at baseline.

d Further adjusted for smoking status (wave 2), diet (wave 2) and exercise (wave 2).

The associations between psychological distress, across the full range of severity, and risk of arthritis and CVD remained significant after additional adjustment for SEP, neighbourhood cohesion, marital status, BMI, baseline disease and health behaviours (Table 2). In the fully adjusted model, the association between distress and risk of COPD remained significant at high levels of distress but not moderate levels of distress. Sobel-Goodman mediation tests showed that smoking partially mediated the associations between psychological distress and risk of incident chronic disease. Smoking status accounted for 4.6% of the total effect of distress on risk of developing arthritis (p = .001), 6.8% of effect on COPD (p = .002), and 2.5% of the effect on CVD (p = .030). Diet and exercise had no significant mediating effects. Bootstrap tests of mediation confirmed these results.

In total, 25% of the participants who had data on incident disease were excluded from our analyses because of missing data on one or more of the covariates. In order to investigate whether excluding these cases biased the results of this study, we repeated analyses using imputed covariate data for participants with complete data on incident disease. The ORs for the sample with imputed covariate data were similar to the ORs for the restricted sample (Table 3). The effect estimates were stronger in the imputed sample for COPD and diabetes, such that psychological distress significantly predicted risk of developing COPD and diabetes at both moderate and high levels of distress in the fully adjusted models (Table 3).

**Table 3:** Odds Ratios (95% CI) for incident chronic disease according to level of psychological distress using imputed covariate data (n = 25,733).

Chronic disease	Level of psychological distress			
	Asymptomatic	Low	Moderate	High
Arthritis	n = 291	n = 213	n = 97	n = 91
Model 1^a^	**1.00**	**1.48** (1.22–1.78)⁎⁎	**1.96** (1.50–2.56)⁎⁎	**2.67** (2.07–3.44)⁎⁎
Model 4^b^	**1.00**	**1.43** (1.18–1.73)⁎⁎	**1.87** (1.43–2.45)⁎⁎	**2.40** (1.85–3.12)⁎⁎

CVD	n = 136	n = 119	n = 43	n = 50
Model 1^a^	**1.00**	**1.75** (1.34–2.27)⁎⁎	**1.88** (1.25–2.81)⁎⁎	**3.22** (2.27–4.57)⁎⁎
Model 4^b^	**1.00**	**1.63** (1.25–2.12)⁎⁎	**1.68** (1.11–2.52)⁎	**2.61** (1.82–3.75)⁎⁎

COPD	n = 45	n = 41	n = 22	n = 26
Model 1^a^	**1.00**	**1.68** (1.09–2.60)⁎	**2.48** (1.40–4.41)⁎⁎	**4.37** (2.66–7.20)⁎⁎
Model 4^b^	**1.00**	**1.52** (0.98–2.36)	**2.03** (1.13–3.65)⁎	**3.18** (1.89–5.36)⁎⁎

Diabetes	n = 134	n = 75	n = 47	n = 39
Model 1^a^	**1.00**	**1.03** (0.75–1.41)	**1.83** (1.25–2.67)⁎⁎	**2.09** (1.42–3.08)⁎⁎
Model 4^b^	**1.00**	**0.92** (0.67–1.26)	**1.63** (1.10–2.42)⁎	**1.60** (1.07–2.39)⁎

⁎ p < .05.

⁎⁎ p < .01.

a Adjusted for age and sex.

b Further adjusted for SEP (wave 1), neighbourhood cohesion (wave 1), marital status (wave 1), BMI (wave 1), other chronic diseases at baseline, smoking status (wave 2), diet (wave 2) and exercise (wave 2).

We examined whether the associations between psychological distress and chronic disease incidence varied according to socioeconomic position after controlling for age, sex, BMI and disease at baseline. We found that the relationship between psychological distress and risk of diabetes differs by SEP (OR 1.14; 1.01–1.29; p = .034) such that the effect of distress is significant in the manual group (OR 1.10; 1.02–1.18; p = .012) but not in the non-manual group (OR 0.96; 0.97–1.06; p = .40). All other interactions were not significant (p > .10) (results not shown).

## 6 Discussion

In this sample of 16,485 UK adults, higher levels of psychological distress were associated with increased risk of developing arthritis, COPD and CVD over a 3-year follow-up period. There were no significant associations between distress and risk of incident diabetes. Psychological distress was found to increase risk of incident arthritis, COPD and CVD across the whole range of symptom severity (i.e. low, moderate and high distress) in a dose-response pattern although the effects of low distress on risk of COPD were not significant. The strength of the associations between low, moderate and high distress and incidence of arthritis and CVD was only partially attenuated after full adjustment for covariates. The association between moderate distress and risk of COPD became non-significant after adjusting for all potential confounding or mediating variables, but that between high distress and risk of COPD persisted. Mediation tests showed that the effects of psychological distress on risk of incident arthritis, COPD and CVD were partially mediated through smoking status at baseline. Diet and exercise had no significant mediating effects. Our analyses using data with imputed covariate values led to similar ORs for arthritis and CVD and stronger effect estimates for COPD and diabetes. This suggests that restricting our sample to complete case resulted in us underestimating the true associations between distress and risk of developing COPD and diabetes. Perhaps this is because participants with poorer health and higher levels of distress are less likely to provide complete data [[36], [37], [38]], thus introducing a bias in our restricted sample towards people who are healthy and less distressed.

We found that the effect of psychological distress on risk of diabetes—but not on risk of the other chronic diseases studied—differed according to SEP, such that the effect was significant in people who had a manual SEP but not in those who had a non-manual SEP. However, the number of incident cases of diabetes was very small. There were as few as 13 new diabetes diagnoses in people with high distress in the manual group and 6 in people with moderate distress in the non-manual group, which is likely to lead to positive or negative bias [39]. We therefore conclude that there is no convincing evidence to suggest that the effects of psychological distress on risk of arthritis, CVD, COPD and diabetes vary according to SEP.

To the best of our knowledge, this the first longitudinal study to examine the relationship between incident arthritis, COPD, CVD and diabetes and psychological distress across the whole range of distress symptoms. Previous longitudinal studies have found that clinical depression – or high psychological distress (GHQ ≥ 4) – increases risk of arthritis [1], COPD [19] and CVD [7,8]. Here, we found a dose-response relationship between distress and risk of developing arthritis, COPD and CVD, which is consistent with studies which found a dose-response relationship between distress and mortality from cardiovascular disease and all causes [22,25].

We found no significant associations between psychological distress and risk of diabetes at low, moderate or high levels of psychological distress. Contrary to these findings, a meta-analysis of nine longitudinal studies found that clinical depression significantly increases risk of type 2 diabetes [10]. In another study, risk of developing type 2 diabetes was found to increase incrementally with increasing severity of depressive symptoms [40]. We repeated analyses with imputed covariate data for incomplete cases and found that risk of developing diabetes was significantly increased by moderate distress (OR 1.63; 1.10–2.42) and high distress (OR 1.60; 1.07–2.39) after adjusting for all potential confounding. This suggests that restricting our sample to complete cases is likely to be responsible for the discrepancy between results.

Previous studies have shown a stronger association between distress and mortality in people of lower socioeconomic position [22,25]. To our knowledge, it is not known whether there is a similar interaction between psychological distress and SEP as regards risk of chronic diseases. Here, we did not find evidence to suggest that the effects of psychological distress on the risk of incident arthritis, COPD and CVD vary according to SEP. It may be the case that the effects of distress vary according to SEP for prognosis of these diseases (i.e. mortality) but not onset of disease. We found that the effect of distress on risk of diabetes varied according to SEP but case numbers were very small. This study was underpowered to assess moderation and these negative findings could be due to type II error.

In this study, we found that the effects of distress on risk of developing arthritis, CVD or COPD were partially mediated by smoking status. This may reflect the fact that distressed individuals smoke to cope with or relieve psychological distress [41,42]. In this sample, people with high levels of distress were more likely to smoke (Table 1) but, despite this, smoking only explained 4.6%, 6.8%, and 2.5% of the effect of distress on risk of arthritis, COPD, or CVD respectively.

Another possible explanation for the increased risk of arthritis, COPD and CVD associated with increasing levels of distress is the link between distress and inflammation. Chronic psychological distress leads to dysregulation of the hypothalamic-pituitary-adrenal axis and increased cortisol levels [43,44]. These changes bring about a heightened inflammatory response across the whole body [45,46], which is known to increase risk of arthritis [47], COPD hospitalisations [48] and CHD events [49,50]. The precise mechanisms of these associations are not clear but chronic inflammation is likely to increase disease risk by leading to hypertension, raised heart rate, raised cholesterol, insulin resistance, endothelial dysfunction and deposition of fat in the abdomen [43,44,51]. Future research should examine the mediating role of inflammatory markers.

Finally, the possibility of surveillance bias must not be overlooked. Higher rates of disease diagnosis in people with high distress may reflect the fact that people who have depression are more likely to consult their doctor and receive a diagnosis [52].

This study included a large sample (N = 16,485) that was highly representative of the UK adult population. The sample was culturally diverse and spanned the entire adult age range. Data was gathered by highly experienced interviewers who received extensive training to ensure all participants were interviewed in the same way [53]. However, our study also has a number of limitations associated with data collection. First, baseline disease cases and incident disease cases were determined using self-report of diagnoses and not objective medical records. However, there is generally a strong agreement between self-reports and medical records [54,55]. Second, the interview questions did not distinguish between different types of arthritis, diabetes and stroke. The different forms of each disease result from distinct pathological processes which may have different relationships with distress. Third, data on smoking, diet and exercise were gathered in wave 2 and not at baseline. However, longitudinal research provides evidence that engagement in these behaviours tends to remain stable over a period of 4 years [56,57]. Fourth, neighbourhood cohesion may not provide an accurate measure of social support in the UK because most significant social relationships occur beyond the local neighbourhood [58].

There were also limitations relating to statistical analysis and interpretation. First, a substantial proportion of participants (58.3%) were excluded from analyses due to missing data. To investigate the effect of this, we compared the results of our analyses with results using imputed covariate data and found that the bias introduced by restricting the sample led to an underestimation of effect sizes. Second, previous studies have found that the strengths of the associations between distress and incident disease differed between sexes (e.g. 19, 20, 45). We also conducted preliminary analyses by sex but there was a very low number of disease events in some groups so the results were vulnerable to bias [39]. Third, the possibility of reverse causality must not be overlooked. Psychological distress is a common consequence of COPD [59] and arthritis [60] in particular. This study excluded people who had disease at baseline but undiagnosed disease could give rise to symptoms of distress that feature in the GHQ-12 (e.g. loss of sleep). There can be significant delays between the onset of symptoms and diagnosis of arthritis [61] and COPD [62,63] so high levels of distress may be a consequence of undiagnosed disease processes. The potential influence of reverse causality could be minimised by following participants for a longer period of time and excluding disease events in the first two years of follow-up.

With the limitations discussed in mind, we conclude that psychological distress increases risk of developing arthritis, COPD and CVD in a dose-response pattern. These relationships are partially mediated by smoking status. There was no evidence for graded associations between distress and risk of diabetes. However, distress significantly increased risk of diabetes in manual SEP albeit the number of cases were very small. We found no evidence that the strength of associations varied according to SEP for arthritis, COPD and CVD. These findings have considerable clinical and public health implications. First, screening for distress may help to identify those at risk of developing arthritis, COPD and CVD. Second, interventions to improve distress may help to prevent and limit progression of disease, even for people with low levels of distress. A number of meta-analyses have found that psychological interventions serve to decrease pain and joint swelling in arthritis [64], improve exercise capacity in COPD [65] and reduce risk of recurrent cardiac events [66]. Our findings have particular significance for primary healthcare physicians as they have a leading role in preventing and managing chronic disease [67] and in diagnosing and managing psychological distress in patients [68,69]. Our findings are also highly relevant to the whole UK population since arthritis, COPD and CVD are among the most common causes of disability and death in UK adults [70]. Future longitudinal studies should examine the effects of psychological distress on risk of chronic disease over a longer follow-up period and investigate the mediating role of inflammatory biomarkers.

## Conflicts of interest and sources of funding

The authors have no conflicts of interest to declare.
